# Agricultural College

**Published:** 1856-01

**Authors:** 


Agricultural College.—The *Ohio Agricultural College*, formerly at Oberlin, has been removed to Cleveland, Ohio. We see no reason why young men should not be educated for the plough, as well as for any other occupation. Comparatively speaking, few farmers become rich, not, however, because it is not a highly remunerative calling, but because ignorant men without capital so generally embark in it. If a man is rich and has a practical as well as a theoretical agricultural education, with a love for his business, there is no occupation in the country which will yield a larger or more certain dividend, not only in money but in health, and years, and honor.

